# (*S*,*S*)-Di­iodido­{3,3′-methyl­enebis[1-(1-hy­droxy-4-methyl­pentan-2-yl)imidazol-2-ylene]}palladium(II) ethanol monosolvate

**DOI:** 10.1107/S2414314625004237

**Published:** 2025-06-10

**Authors:** Ping Liao, Yanping Wang, Li Zhong, Jinwei Yuan, Liangru Yang

**Affiliations:** a School of Chemistry & Chemical Engineering Henan University of Technology,Zhengzhou 450001 People’s Republic of China; b School of Energy and Chemical Engineering, Puyang Vocational and Technical, College, Puyang 457001, People’s Republic of China; Purdue University, USA

**Keywords:** crystal structure, X-ray crystallography, *N*-heterocyclic carbene palladium, chelating

## Abstract

The structure of the title solvated bis-*N*-heterocyclic carbene palladium(II) complex, [PdI_2_(C_19_H_32_N_4_O_2_)_2_·C_2_H_6_O, exhibits a slightly distorted square-planar coord­in­ation at the palladium(II) atom, with the six-membered chelate ring adopting a boat conformation. One disordered ethanol solvent mol­ecule was co-crystallized. There are inter­molecular O—H⋯O hydrogen bonds in the structure. The chiral centers of the precursor imidazolium salt was preserved during the metalation process.

## Abstract 2

The title methyl­ene-bridged bis-*N*-heterocyclic carbene (NHC) palladium complex exhibits a distorted square-planar geometry around the palladium center, with the six-membered chelate ring adopting a boat conformation.

## Graphical abstract

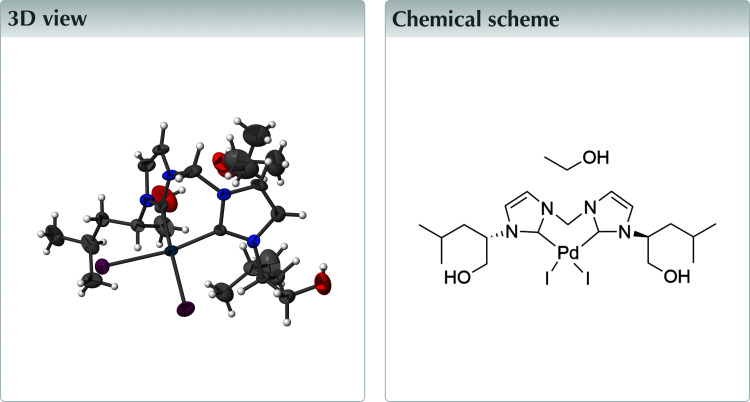

## Structure description

Palladium complexes supported by *N*-heterocyclic carbene (NHC) ligands have become indispensable catalysts in organometallic chemistry owing to their remarkable thermal stability, adjustable electronic characteristics, and broad reactivity (Fortman & Nolan, 2011 ▸; Hopkinson *et al.*, 2014 ▸). Methyl­ene-bridged bidentate chelating bis-NHC ligands stand out in this context, as their rigid architecture introduces geometric constraints that reinforce ligand durability while fine-tuning the electronic configuration of the palladium center – a feature critical for advancing cross-coupling, C—H activation, and other industrially vital catalytic processes (Liu *et al.* 2018 ▸; Gardiner & Ho, 2018 ▸). Here we present the structural characterization *via* single-crystal X-ray diffraction of a palladium complex chelated by a methyl­ene-bridged bidentate NHC ligand system. The chiral centers of the precursor imidazolium salt was preserved during the metalation process.

**Figure 1:**
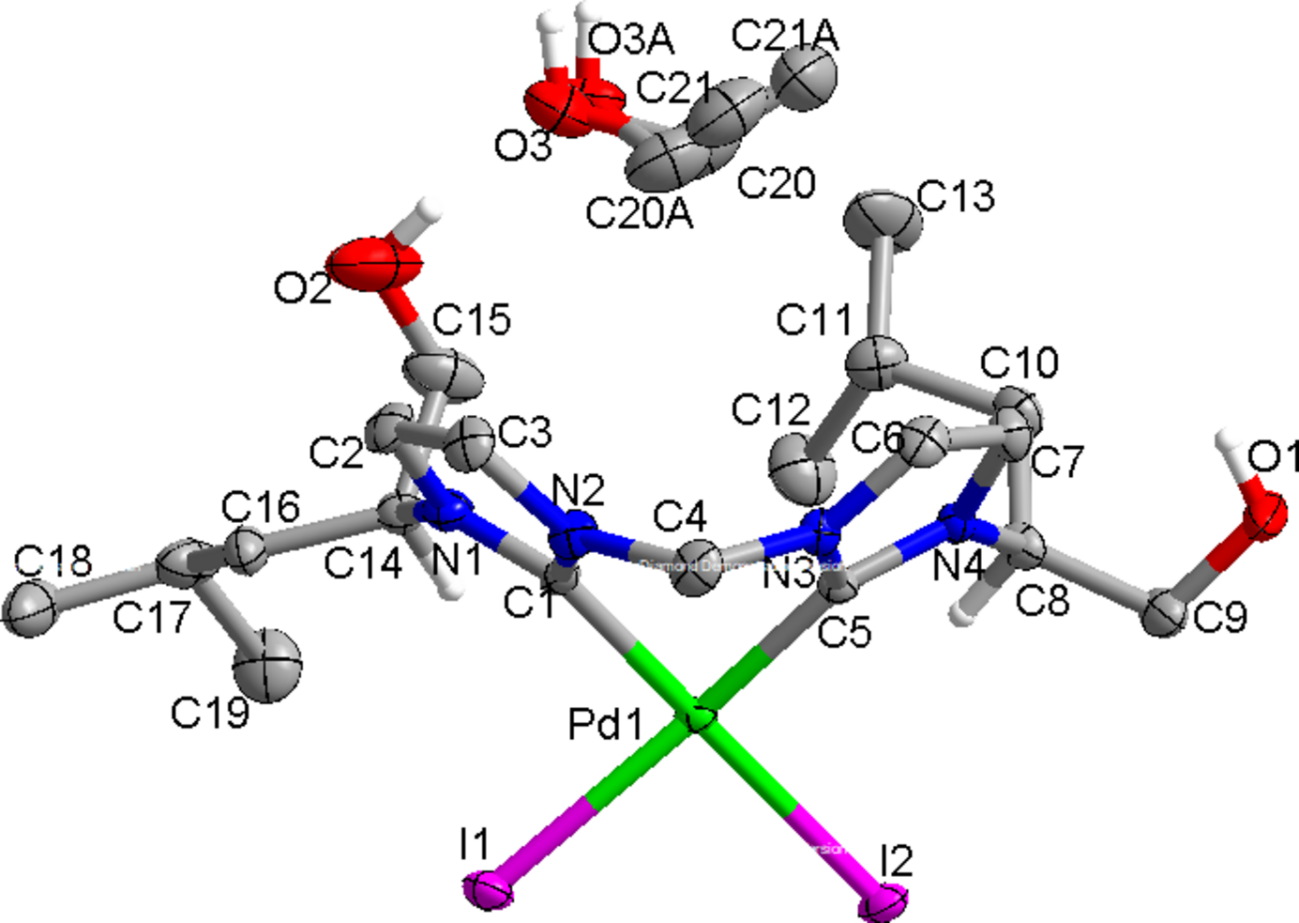
The mol­ecular structure of the title complex, shown with 50% probability displacement ellipsoids. Carbon bound H-atoms other than at chiral centers are omitted for clarity.

**Figure 2:**
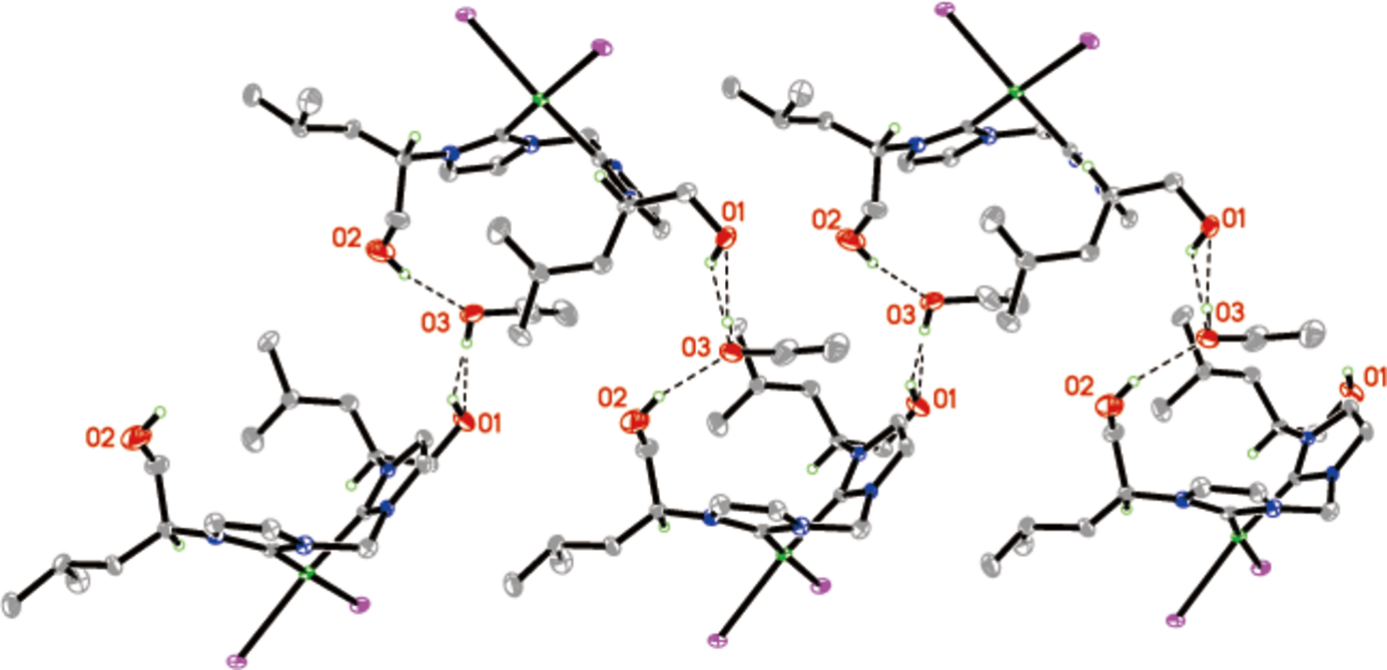
The O—H⋯O inter­actions in the structure. [Symmetry codes: (i) *x* + 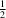, −*y* + 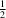, −*z* + 1; (ii) *x* − 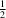, −*y* + 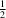, −*z* + 1].

**Figure 3:**
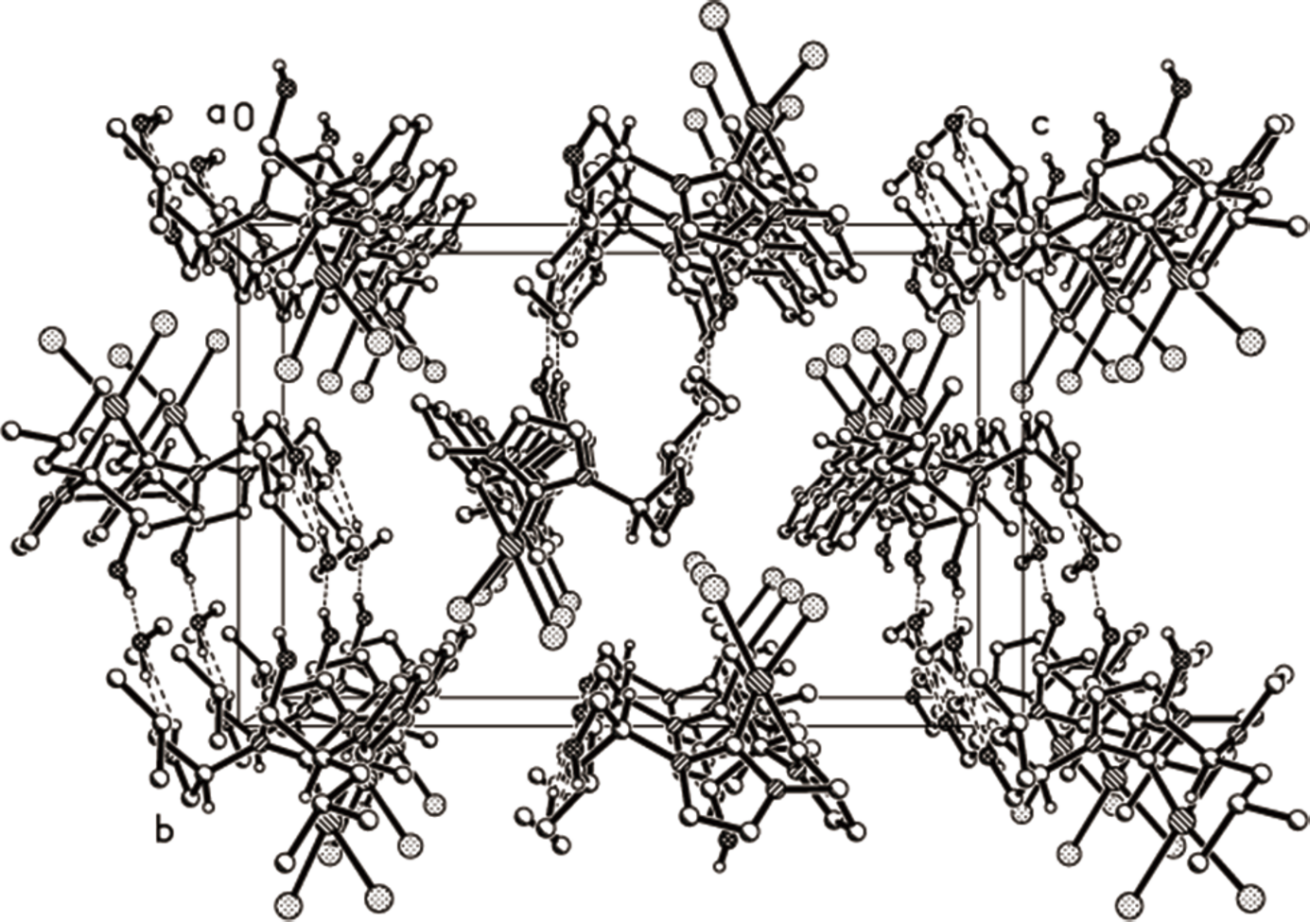
The crystallographic packing visualization of the structure along the *a* axis direction.

In the title complex (Fig. 1 ▸), the palladium(II) atom is coordinated by the two carbene C-atoms C1 and C5, and by the two iodine atoms I1, and I2, resulting in a slightly distorted square-planar coordination. The Pd1—C1, Pd1—C5, Pd1—I1 and Pd1—I2 bond lengths are 2.003 (10), 1.997 (10), 2.6518 (10), and 2.6501 (10) Å, respectively. The six-membered chelate ring (C1/N2/C4/N3/C5/Pd1) adopts a boat conformation, with the torsion angles C4—N2—C1—Pd1 and C4—N3—C5—Pd1 being 10.1 (14) and −1.5 (13)°, respectively. The bond angles N2—C4—N3 and C1—Pd1—C5 are 108.8 (9) and 83.7 (4) Å respectively. In the crystal, inter­molecular O—H⋯O hydrogen bonds occur (Table 1 ▸, Fig. 2 ▸). The packing is shown in Fig. 3 ▸.

## Synthesis and crystallization

A mixture of 3,3′-methyl­enebis[1-(1-hy­droxy-4-methyl­pentan-2-yl)-1*H*-imidazolium] diiodide (2 mmol, 1.21 g) (Meng, 2023 ▸) and Pd(OAc)_2_ (2 mmol, 0.45 g) was stirred in CH_3_CN (15 mL) at 80°C for 18 h. The reaction mixture was then concentrated. Purification of the residue by column chromatography (silica, CH_2_Cl_2_/acetone, gradient elution, 3:1–1:1 *v*/*v*) produced the title NHC palladium complex as a yellow solid (0.81 g, 57%). Crystallization of the solids from a CH_2_Cl_2_/ethanol/hexane solution afforded the title complex as yellow crystals. HR–MS (ESI) *m*/*z* calculated for C_19_H_32_IN_4_O_2_Pd^+^ [*M* - I]^+^ 581.0605, found 581.0606. ^1^H NMR (400 MHz, DMSO-*d*_6_): δ 7.61–7.39 (*m*, 4H), 6.28 (*d*, *J* = 24.8 Hz, 2H), 5.33–5.05 (*m*, 3H), 3.81–3.72 (*m*, 3H), 3.37 (*s*, 1H), 1.91–1.21 (*m*, 6H), 1.03- 0.78 (*m*, 12H) p.p.m.. ^13^C NMR (100 MHz, DMSO-d_6_): δ 162.8, 161.7, 122.6, 121.4, 120.4, 63.0, 60.9, 60.2, 25.0, 24.5, 23.1, 23.0, 22.5 p.p.m..

## Refinement

Crystal data, data collection and structure refinement details are summarized in Table 2 ▸. The ethanol solvate mol­ecule was refined as disordered over two orientations. Equivalent bond distances in the major and minor moiety were restrained to be similar (SADI restraints with an e.s.d. of 0.02 Å) and ethanol O—C and C—C bonds were restrained to expected target values of 1.40 (2) and 1.54 (2) Å, respectively. *U^ij^* components of ADPs for disordered atoms closer to each other than 1.7 Å were restrained to be similar within an e.s.d. of 0.01 Å^2^, and to be close to isotropic (e.s.d. 0.01 Å^2^). Subject to these conditions the occupancy ratio refined to 0.58 (4) to 0.42 (4).

## Supplementary Material

Crystal structure: contains datablock(s) I. DOI: 10.1107/S2414314625004237/zl4080sup1.cif

Structure factors: contains datablock(s) I. DOI: 10.1107/S2414314625004237/zl4080Isup3.hkl

CCDC reference: 2312033

Additional supporting information:  crystallographic information; 3D view; checkCIF report

## Figures and Tables

**Table 1:** Hydrogen-bond geometry (Å, °)

*D*—H⋯*A*	*D*—H	H⋯*A*	*D*⋯*A*	*D*—H⋯*A*
O1—H1⋯O3^i^	0.82	2.21	2.85 (5)	134
O1—H1⋯O3*A*^i^	0.82	2.13	2.80 (6)	140
O2—H2⋯O3	0.82	2.06	2.86 (5)	164
O2—H2⋯O3*A*	0.82	2.09	2.90 (7)	171
O3—H3*A*⋯O1^ii^	0.82	2.04	2.85 (5)	168
O3*A*—H3*AA*⋯O1^ii^	0.82	1.99	2.80 (6)	170

Symmetry codes: (i) 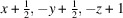; (ii) 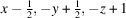.

**Table 2:** Experimental details

Crystal data	
Chemical formula	[PdI_2_(C_19_H_32_N_4_O_2_)]·C_2_H_6_O
*M*_r_	754.75
Crystal system, space group	Orthorhombic, *P*2_1_2_1_2_1_
Temperature (K)	293
*a*, *b*, *c* (Å)	11.5190 (2), 12.4798 (2), 19.5537 (3)
*V* (Å^3^)	2810.92 (8)
*Z*	4
Radiation type	Cu *K*α
μ (mm^−1^)	22.79
Crystal size (mm)	0.15 × 0.12 × 0.10

Data collection	
Diffractometer	Xcalibur, Eos, Gemini
Absorption correction	Multi-scan (*CrysAlis PRO*; Rigaku OD, 2019 ▸)
*T*_min_, *T*_max_	0.581, 1.000
No. of measured, independent and observed [*I* > 2σ(*I*)] reflections	10617, 5265, 4887
*R*_int_	0.045
(sin θ/λ)_max_ (Å^−1^)	0.613

Refinement	
*R*[*F*^2^ > 2σ(*F*^2^)], *wR*(*F*^2^), *S*	0.040, 0.099, 1.04
No. of reflections	5265
No. of parameters	316
No. of restraints	70
H-atom treatment	H-atom parameters constrained
Δρ_max_, Δρ_min_ (e Å^−3^)	0.94, −0.78
Absolute structure	Flack *x* determined using 1903 quotients [(*I*^+^)−(*I*^−^)]/[(*I*^+^)+(*I*^−^)] (Parsons *et al.*, 2013 ▸)
Absolute structure parameter	−0.004 (8)

Computer programs: *CrysAlis PRO* (Rigaku OD, 2019 ▸), *SHELXS* (Sheldrick, 2008 ▸), *SHELXL2019/3* (Sheldrick, 2015 ▸) and *OLEX2* (Dolomanov *et al.*, 2009 ▸).

## full crystallographic data

### (S,S)-Diiodido{3,3'-methylenebis[1-(1-hydroxy-4-methylpentan-2-yl)imidazol-2-ylene]}palladium(II) ethanol monosolvate Crystal data

**Table:**

[PdI_2_(C_19_H_32_N_4_O_2_)]·C_2_H_6_O	*D*_x_ = 1.783 Mg m^−^^3^
*M**_r_* = 754.75	Cu *K*α radiation, λ = 1.54184 Å
Orthorhombic, *P*2_1_2_1_2_1_	Cell parameters from 4920 reflections
*a* = 11.5190 (2) Å	θ = 4.2–70.9°
*b* = 12.4798 (2) Å	µ = 22.79 mm^−^^1^
*c* = 19.5537 (3) Å	*T* = 293 K
*V* = 2810.92 (8) Å^3^	Block, yellow
*Z* = 4	0.15 × 0.12 × 0.1 mm
*F*(000) = 1472	

### (S,S)-Diiodido{3,3'-methylenebis[1-(1-hydroxy-4-methylpentan-2-yl)imidazol-2-ylene]}palladium(II) ethanol monosolvate Data collection

**Table:**

Xcalibur, Eos, Gemini diffractometer	5265 independent reflections
Radiation source: fine-focus sealed X-ray tube, Enhance (Cu) X-ray Source	4887 reflections with *I* > 2σ(*I*)
Graphite monochromator	*R*_int_ = 0.045
Detector resolution: 16.2312 pixels mm^-1^	θ_max_ = 71.0°, θ_min_ = 4.2°
ω scans	*h* = −12→13
Absorption correction: multi-scan (CrysAlisPro; Rigaku OD, 2019)	*k* = −14→15
*T*_min_ = 0.581, *T*_max_ = 1.000	*l* = −14→23
10617 measured reflections	

### (S,S)-Diiodido{3,3'-methylenebis[1-(1-hydroxy-4-methylpentan-2-yl)imidazol-2-ylene]}palladium(II) ethanol monosolvate Refinement

**Table:**

Refinement on *F*^2^	Hydrogen site location: inferred from neighbouring sites
Least-squares matrix: full	H-atom parameters constrained
*R*[*F*^2^ > 2σ(*F*^2^)] = 0.040	*w* = 1/[σ^2^(*F*_o_^2^) + (0.0424*P*)^2^] where *P* = (*F*_o_^2^ + 2*F*_c_^2^)/3
*wR*(*F*^2^) = 0.099	(Δ/σ)_max_ = 0.001
*S* = 1.04	Δρ_max_ = 0.94 e Å^−^^3^
5265 reflections	Δρ_min_ = −0.78 e Å^−^^3^
316 parameters	Absolute structure: Flack *x* determined using 1903 quotients [(*I*^+^)-(*I*^-^)]/[(*I*^+^)+(*I*^-^)] (Parsons *et al.*, 2013)
70 restraints	Absolute structure parameter: −0.004 (8)
Primary atom site location: structure-invariant direct methods	

### (S,S)-Diiodido{3,3'-methylenebis[1-(1-hydroxy-4-methylpentan-2-yl)imidazol-2-ylene]}palladium(II) ethanol monosolvate Special details

**Table:**

Geometry. All esds (except the esd in the dihedral angle between two l.s. planes) are estimated using the full covariance matrix. The cell esds are taken into account individually in the estimation of esds in distances, angles and torsion angles; correlations between esds in cell parameters are only used when they are defined by crystal symmetry. An approximate (isotropic) treatment of cell esds is used for estimating esds involving l.s. planes.

### (S,S)-Diiodido{3,3'-methylenebis[1-(1-hydroxy-4-methylpentan-2-yl)imidazol-2-ylene]}palladium(II) ethanol monosolvate Fractional atomic coordinates and isotropic or equivalent isotropic displacement parameters (Å^2^)

**Table:**

	*x*	*y*	*z*	*U*_iso_*/*U*_eq_	Occ. (<1)
I1	0.22094 (7)	0.76030 (6)	0.28882 (4)	0.03677 (18)	
I2	0.47860 (7)	0.80151 (6)	0.40700 (4)	0.03859 (19)	
Pd1	0.36900 (7)	0.63403 (5)	0.35432 (3)	0.02394 (16)	
O1	0.7132 (10)	0.5393 (7)	0.5616 (7)	0.066 (3)	
H1	0.687748	0.478394	0.557033	0.100*	
O2	0.0396 (14)	0.3230 (10)	0.3847 (11)	0.111 (6)	
H2	0.088367	0.278229	0.395532	0.167*	
O3	0.231 (4)	0.179 (4)	0.3972 (18)	0.067 (9)	0.58 (4)
H3A	0.219942	0.114842	0.403898	0.101*	0.58 (4)
N1	0.1911 (9)	0.4636 (8)	0.3073 (5)	0.035 (2)	
N2	0.3651 (9)	0.4424 (7)	0.2701 (4)	0.033 (2)	
N3	0.5322 (8)	0.4567 (7)	0.3395 (4)	0.0265 (18)	
N4	0.5416 (8)	0.5034 (7)	0.4443 (4)	0.0269 (18)	
C1	0.2992 (9)	0.5057 (8)	0.3086 (5)	0.027 (2)	
C2	0.1928 (12)	0.3735 (10)	0.2642 (7)	0.045 (3)	
H2A	0.129794	0.330227	0.253169	0.053*	
C3	0.3011 (11)	0.3617 (10)	0.2422 (6)	0.040 (3)	
H3	0.328157	0.308308	0.213123	0.048*	
C4	0.4900 (12)	0.4538 (10)	0.2692 (6)	0.040 (3)	
H4A	0.511298	0.519395	0.245733	0.048*	
H4B	0.524699	0.393992	0.245039	0.048*	
C5	0.4888 (9)	0.5291 (8)	0.3855 (5)	0.024 (2)	
C6	0.6090 (10)	0.3899 (9)	0.3703 (6)	0.036 (3)	
H6	0.649617	0.334577	0.349275	0.043*	
C7	0.6163 (11)	0.4174 (10)	0.4359 (6)	0.040 (3)	
H7	0.662050	0.385333	0.469340	0.048*	
C8	0.5235 (11)	0.5596 (9)	0.5105 (5)	0.033 (2)	
H8	0.467610	0.617056	0.501538	0.039*	
C9	0.6332 (12)	0.6132 (10)	0.5348 (6)	0.044 (3)	
H9A	0.668642	0.651178	0.496925	0.053*	
H9B	0.613832	0.665412	0.569787	0.053*	
C10	0.4671 (12)	0.4858 (11)	0.5620 (6)	0.043 (3)	
H10A	0.514080	0.421594	0.565568	0.052*	
H10B	0.468800	0.520929	0.606251	0.052*	
C11	0.3444 (12)	0.4526 (11)	0.5477 (7)	0.046 (3)	
H11	0.344444	0.419456	0.502233	0.055*	
C12	0.2626 (15)	0.5474 (14)	0.5439 (10)	0.075 (5)	
H12A	0.187471	0.523464	0.529165	0.112*	
H12B	0.292290	0.598890	0.511917	0.112*	
H12C	0.256257	0.579808	0.588263	0.112*	
C13	0.3018 (15)	0.3686 (18)	0.5970 (12)	0.097 (8)	
H13A	0.310226	0.394318	0.642997	0.146*	
H13B	0.346479	0.304241	0.591359	0.146*	
H13C	0.221440	0.353569	0.588082	0.146*	
C14	0.0911 (10)	0.4990 (9)	0.3473 (6)	0.035 (2)	
H14	0.111652	0.568367	0.367423	0.042*	
C15	0.0739 (16)	0.4208 (14)	0.4066 (10)	0.072 (5)	
H15A	0.146138	0.413779	0.431679	0.086*	
H15B	0.015924	0.449361	0.437636	0.086*	
C16	−0.0123 (10)	0.5180 (10)	0.3007 (7)	0.042 (3)	
H16A	−0.032345	0.450933	0.278620	0.050*	
H16B	0.010273	0.568200	0.265291	0.050*	
C17	−0.1204 (12)	0.5616 (12)	0.3372 (9)	0.055 (4)	
H17	−0.148346	0.506263	0.368686	0.067*	
C18	−0.2155 (13)	0.5807 (12)	0.2826 (11)	0.070 (5)	
H18A	−0.185454	0.626213	0.247224	0.106*	
H18B	−0.281394	0.614412	0.303586	0.106*	
H18C	−0.238542	0.513307	0.263256	0.106*	
C19	−0.0991 (15)	0.6642 (14)	0.3786 (8)	0.069 (5)	
H19A	−0.049281	0.648402	0.416660	0.103*	
H19B	−0.171869	0.691329	0.395168	0.103*	
H19C	−0.062953	0.716958	0.349964	0.103*	
C20	0.348 (4)	0.205 (6)	0.415 (2)	0.090 (11)	0.58 (4)
H20A	0.360606	0.188291	0.463158	0.108*	0.58 (4)
H20B	0.360175	0.281393	0.409172	0.108*	0.58 (4)
C20A	0.345 (5)	0.204 (9)	0.391 (3)	0.091 (12)	0.42 (4)
H20C	0.358128	0.280701	0.386164	0.109*	0.42 (4)
H20D	0.354750	0.170198	0.346588	0.109*	0.42 (4)
C21	0.435 (3)	0.143 (3)	0.371 (2)	0.093 (12)	0.58 (4)
H21A	0.408514	0.141784	0.324648	0.140*	0.58 (4)
H21B	0.440859	0.070551	0.387875	0.140*	0.58 (4)
H21C	0.509430	0.176727	0.373598	0.140*	0.58 (4)
C21A	0.425 (4)	0.155 (4)	0.445 (3)	0.094 (16)	0.42 (4)
H21D	0.427849	0.078544	0.438395	0.141*	0.42 (4)
H21E	0.395236	0.170467	0.489548	0.141*	0.42 (4)
H21F	0.501320	0.184250	0.440102	0.141*	0.42 (4)
O3A	0.232 (6)	0.183 (5)	0.419 (2)	0.061 (11)	0.42 (4)
H3AA	0.227571	0.119314	0.429671	0.091*	0.42 (4)

### (S,S)-Diiodido{3,3'-methylenebis[1-(1-hydroxy-4-methylpentan-2-yl)imidazol-2-ylene]}palladium(II) ethanol monosolvate Atomic displacement parameters (Å^2^)

**Table:**

	*U*^11^	*U*^22^	*U*^33^	*U*^12^	*U*^13^	*U*^23^
I1	0.0490 (4)	0.0298 (3)	0.0316 (3)	0.0014 (3)	−0.0019 (3)	0.0079 (3)
I2	0.0528 (4)	0.0269 (3)	0.0361 (3)	−0.0131 (3)	−0.0041 (3)	−0.0017 (3)
Pd1	0.0329 (4)	0.0195 (3)	0.0194 (3)	−0.0027 (3)	0.0014 (3)	−0.0020 (3)
O1	0.059 (6)	0.034 (5)	0.106 (9)	−0.007 (5)	−0.043 (7)	0.001 (6)
O2	0.109 (12)	0.060 (8)	0.164 (17)	−0.003 (8)	0.025 (12)	0.035 (10)
O3	0.077 (15)	0.058 (14)	0.067 (19)	0.017 (12)	0.005 (16)	0.017 (15)
N1	0.042 (6)	0.027 (4)	0.037 (5)	0.000 (4)	0.001 (4)	−0.002 (4)
N2	0.042 (5)	0.032 (4)	0.024 (4)	0.001 (4)	0.004 (4)	−0.011 (3)
N3	0.025 (4)	0.032 (4)	0.023 (4)	0.002 (4)	0.004 (4)	−0.005 (3)
N4	0.028 (4)	0.028 (4)	0.025 (4)	−0.002 (4)	−0.002 (4)	0.000 (3)
C1	0.034 (6)	0.024 (5)	0.023 (4)	−0.009 (4)	−0.002 (4)	0.001 (4)
C2	0.049 (7)	0.034 (6)	0.051 (7)	−0.008 (6)	−0.006 (6)	−0.014 (6)
C3	0.048 (7)	0.039 (6)	0.032 (5)	0.004 (6)	0.000 (5)	−0.017 (5)
C4	0.048 (7)	0.046 (6)	0.026 (5)	−0.003 (6)	0.007 (5)	−0.012 (5)
C5	0.026 (5)	0.022 (4)	0.024 (4)	0.002 (4)	0.001 (4)	−0.002 (4)
C6	0.031 (6)	0.032 (6)	0.045 (6)	0.009 (5)	0.007 (5)	−0.013 (5)
C7	0.034 (6)	0.045 (6)	0.040 (6)	0.006 (6)	−0.003 (5)	−0.001 (5)
C8	0.046 (6)	0.031 (5)	0.021 (4)	0.007 (5)	0.000 (5)	−0.005 (4)
C9	0.058 (8)	0.035 (6)	0.039 (6)	0.002 (6)	−0.011 (6)	−0.008 (5)
C10	0.051 (7)	0.049 (7)	0.030 (5)	0.004 (6)	−0.001 (6)	0.001 (5)
C11	0.048 (8)	0.047 (7)	0.043 (7)	0.001 (6)	0.015 (6)	−0.002 (6)
C12	0.063 (10)	0.076 (12)	0.085 (13)	0.024 (10)	0.004 (10)	−0.008 (10)
C13	0.061 (11)	0.111 (17)	0.119 (17)	−0.016 (11)	0.018 (12)	0.060 (15)
C14	0.038 (6)	0.029 (5)	0.038 (6)	0.002 (5)	0.000 (5)	−0.002 (5)
C15	0.073 (11)	0.066 (10)	0.076 (11)	0.013 (9)	0.028 (10)	0.024 (9)
C16	0.036 (6)	0.036 (6)	0.053 (7)	0.003 (5)	−0.011 (6)	−0.003 (5)
C17	0.037 (7)	0.047 (7)	0.083 (10)	−0.002 (6)	0.013 (8)	0.014 (7)
C18	0.042 (7)	0.047 (8)	0.122 (15)	0.001 (7)	−0.007 (10)	0.004 (9)
C19	0.071 (11)	0.074 (11)	0.061 (9)	0.012 (9)	0.007 (8)	−0.021 (8)
C20	0.096 (17)	0.069 (14)	0.11 (3)	−0.025 (14)	0.02 (2)	−0.01 (3)
C20A	0.095 (18)	0.072 (15)	0.11 (3)	−0.023 (15)	0.02 (2)	0.00 (3)
C21	0.089 (19)	0.072 (17)	0.12 (2)	−0.026 (16)	0.011 (17)	−0.012 (17)
C21A	0.08 (2)	0.08 (2)	0.12 (3)	−0.013 (19)	−0.02 (2)	0.02 (2)
O3A	0.09 (2)	0.036 (14)	0.05 (2)	0.000 (14)	0.018 (17)	0.007 (15)

### (S,S)-Diiodido{3,3'-methylenebis[1-(1-hydroxy-4-methylpentan-2-yl)imidazol-2-ylene]}palladium(II) ethanol monosolvate Geometric parameters (Å, º)

**Table:**

I1—Pd1	2.6518 (10)	C11—C12	1.514 (19)
I2—Pd1	2.6501 (10)	C11—C13	1.51 (2)
Pd1—C1	2.003 (10)	C12—H12A	0.9600
Pd1—C5	1.997 (10)	C12—H12B	0.9600
O1—H1	0.8200	C12—H12C	0.9600
O1—C9	1.405 (15)	C13—H13A	0.9600
O2—H2	0.8200	C13—H13B	0.9600
O2—C15	1.35 (2)	C13—H13C	0.9600
O3—H3A	0.8200	C14—H14	0.9800
O3—C20	1.43 (2)	C14—C15	1.528 (19)
N1—C1	1.351 (14)	C14—C16	1.518 (16)
N1—C2	1.405 (14)	C15—H15A	0.9700
N1—C14	1.461 (15)	C15—H15B	0.9700
N2—C1	1.329 (14)	C16—H16A	0.9700
N2—C3	1.362 (15)	C16—H16B	0.9700
N2—C4	1.446 (16)	C16—C17	1.534 (18)
N3—C4	1.459 (14)	C17—H17	0.9800
N3—C5	1.369 (12)	C17—C18	1.55 (2)
N3—C6	1.358 (15)	C17—C19	1.53 (2)
N4—C5	1.340 (13)	C18—H18A	0.9600
N4—C7	1.385 (15)	C18—H18B	0.9600
N4—C8	1.487 (12)	C18—H18C	0.9600
C2—H2A	0.9300	C19—H19A	0.9600
C2—C3	1.327 (18)	C19—H19B	0.9600
C3—H3	0.9300	C19—H19C	0.9600
C4—H4A	0.9700	C20—H20A	0.9700
C4—H4B	0.9700	C20—H20B	0.9700
C6—H6	0.9300	C20—C21	1.53 (3)
C6—C7	1.330 (16)	C20A—H20C	0.9700
C7—H7	0.9300	C20A—H20D	0.9700
C8—H8	0.9800	C20A—C21A	1.53 (3)
C8—C9	1.507 (17)	C20A—O3A	1.44 (3)
C8—C10	1.511 (16)	C21—H21A	0.9600
C9—H9A	0.9700	C21—H21B	0.9600
C9—H9B	0.9700	C21—H21C	0.9600
C10—H10A	0.9700	C21A—H21D	0.9600
C10—H10B	0.9700	C21A—H21E	0.9600
C10—C11	1.499 (19)	C21A—H21F	0.9600
C11—H11	0.9800	O3A—H3AA	0.8200
			
I2—Pd1—I1	91.46 (3)	C11—C12—H12C	109.5
C1—Pd1—I1	90.1 (3)	H12A—C12—H12B	109.5
C1—Pd1—I2	174.5 (3)	H12A—C12—H12C	109.5
C5—Pd1—I1	168.9 (3)	H12B—C12—H12C	109.5
C5—Pd1—I2	94.0 (3)	C11—C13—H13A	109.5
C5—Pd1—C1	83.7 (4)	C11—C13—H13B	109.5
C9—O1—H1	109.5	C11—C13—H13C	109.5
C15—O2—H2	109.5	H13A—C13—H13B	109.5
C20—O3—H3A	109.5	H13A—C13—H13C	109.5
C1—N1—C2	108.1 (10)	H13B—C13—H13C	109.5
C1—N1—C14	126.8 (10)	N1—C14—H14	107.0
C2—N1—C14	125.0 (10)	N1—C14—C15	108.5 (11)
C1—N2—C3	110.9 (10)	N1—C14—C16	110.1 (10)
C1—N2—C4	121.1 (9)	C15—C14—H14	107.0
C3—N2—C4	127.3 (10)	C16—C14—H14	107.0
C5—N3—C4	120.9 (9)	C16—C14—C15	116.9 (12)
C6—N3—C4	128.4 (10)	O2—C15—C14	112.0 (16)
C6—N3—C5	110.6 (8)	O2—C15—H15A	109.2
C5—N4—C7	111.5 (9)	O2—C15—H15B	109.2
C5—N4—C8	124.8 (9)	C14—C15—H15A	109.2
C7—N4—C8	123.7 (9)	C14—C15—H15B	109.2
N1—C1—Pd1	133.6 (8)	H15A—C15—H15B	107.9
N2—C1—Pd1	119.9 (8)	C14—C16—H16A	108.7
N2—C1—N1	106.5 (9)	C14—C16—H16B	108.7
N1—C2—H2A	126.4	C14—C16—C17	114.4 (12)
C3—C2—N1	107.2 (11)	H16A—C16—H16B	107.6
C3—C2—H2A	126.4	C17—C16—H16A	108.7
N2—C3—H3	126.4	C17—C16—H16B	108.7
C2—C3—N2	107.3 (10)	C16—C17—H17	108.0
C2—C3—H3	126.4	C16—C17—C18	108.0 (14)
N2—C4—N3	108.8 (9)	C18—C17—H17	108.0
N2—C4—H4A	109.9	C19—C17—C16	114.3 (12)
N2—C4—H4B	109.9	C19—C17—H17	108.0
N3—C4—H4A	109.9	C19—C17—C18	110.4 (13)
N3—C4—H4B	109.9	C17—C18—H18A	109.5
H4A—C4—H4B	108.3	C17—C18—H18B	109.5
N3—C5—Pd1	119.0 (7)	C17—C18—H18C	109.5
N4—C5—Pd1	137.1 (7)	H18A—C18—H18B	109.5
N4—C5—N3	103.9 (8)	H18A—C18—H18C	109.5
N3—C6—H6	125.9	H18B—C18—H18C	109.5
C7—C6—N3	108.1 (10)	C17—C19—H19A	109.5
C7—C6—H6	125.9	C17—C19—H19B	109.5
N4—C7—H7	127.0	C17—C19—H19C	109.5
C6—C7—N4	105.9 (11)	H19A—C19—H19B	109.5
C6—C7—H7	127.0	H19A—C19—H19C	109.5
N4—C8—H8	106.4	H19B—C19—H19C	109.5
N4—C8—C9	111.6 (10)	O3—C20—H20A	109.4
N4—C8—C10	110.7 (9)	O3—C20—H20B	109.4
C9—C8—H8	106.4	O3—C20—C21	111 (4)
C9—C8—C10	114.9 (10)	H20A—C20—H20B	108.0
C10—C8—H8	106.4	C21—C20—H20A	109.4
O1—C9—C8	112.1 (10)	C21—C20—H20B	109.4
O1—C9—H9A	109.2	H20C—C20A—H20D	109.2
O1—C9—H9B	109.2	C21A—C20A—H20C	111.4
C8—C9—H9A	109.2	C21A—C20A—H20D	111.4
C8—C9—H9B	109.2	O3A—C20A—H20C	111.4
H9A—C9—H9B	107.9	O3A—C20A—H20D	111.4
C8—C10—H10A	108.1	O3A—C20A—C21A	102 (6)
C8—C10—H10B	108.1	C20—C21—H21A	109.5
H10A—C10—H10B	107.3	C20—C21—H21B	109.5
C11—C10—C8	116.7 (11)	C20—C21—H21C	109.5
C11—C10—H10A	108.1	H21A—C21—H21B	109.5
C11—C10—H10B	108.1	H21A—C21—H21C	109.5
C10—C11—H11	106.6	H21B—C21—H21C	109.5
C10—C11—C12	112.3 (13)	C20A—C21A—H21D	109.5
C10—C11—C13	112.3 (13)	C20A—C21A—H21E	109.5
C12—C11—H11	106.6	C20A—C21A—H21F	109.5
C13—C11—H11	106.6	H21D—C21A—H21E	109.5
C13—C11—C12	111.8 (14)	H21D—C21A—H21F	109.5
C11—C12—H12A	109.5	H21E—C21A—H21F	109.5
C11—C12—H12B	109.5	C20A—O3A—H3AA	109.5
			
N1—C2—C3—N2	−0.5 (15)	C5—N3—C6—C7	0.2 (14)
N1—C14—C15—O2	−67.9 (18)	C5—N4—C7—C6	0.1 (14)
N1—C14—C16—C17	−176.7 (10)	C5—N4—C8—C9	−116.1 (11)
N3—C6—C7—N4	−0.2 (14)	C5—N4—C8—C10	114.7 (12)
N4—C8—C9—O1	−75.5 (13)	C6—N3—C4—N2	121.1 (12)
N4—C8—C10—C11	−67.5 (14)	C6—N3—C5—Pd1	−177.8 (7)
C1—N1—C2—C3	1.6 (15)	C6—N3—C5—N4	−0.2 (12)
C1—N1—C14—C15	−102.5 (14)	C7—N4—C5—Pd1	177.0 (9)
C1—N1—C14—C16	128.4 (11)	C7—N4—C5—N3	0.0 (12)
C1—N2—C3—C2	−0.8 (14)	C7—N4—C8—C9	63.7 (14)
C1—N2—C4—N3	49.6 (14)	C7—N4—C8—C10	−65.5 (14)
C2—N1—C1—Pd1	178.1 (9)	C8—N4—C5—Pd1	−3.2 (17)
C2—N1—C1—N2	−2.0 (13)	C8—N4—C5—N3	179.9 (9)
C2—N1—C14—C15	72.3 (16)	C8—N4—C7—C6	−179.7 (11)
C2—N1—C14—C16	−56.8 (15)	C8—C10—C11—C12	−60.2 (16)
C3—N2—C1—Pd1	−178.4 (8)	C8—C10—C11—C13	172.7 (14)
C3—N2—C1—N1	1.8 (13)	C9—C8—C10—C11	165.1 (11)
C3—N2—C4—N3	−120.4 (12)	C10—C8—C9—O1	51.5 (15)
C4—N2—C1—Pd1	10.1 (14)	C14—N1—C1—Pd1	−6.3 (18)
C4—N2—C1—N1	−169.7 (10)	C14—N1—C1—N2	173.5 (10)
C4—N2—C3—C2	170.0 (12)	C14—N1—C2—C3	−174.0 (11)
C4—N3—C5—Pd1	−1.5 (13)	C14—C16—C17—C18	177.8 (11)
C4—N3—C5—N4	176.1 (10)	C14—C16—C17—C19	54.5 (17)
C4—N3—C6—C7	−175.7 (12)	C15—C14—C16—C17	59.0 (16)
C5—N3—C4—N2	−54.5 (14)	C16—C14—C15—O2	57.3 (19)

### (S,S)-Diiodido{3,3'-methylenebis[1-(1-hydroxy-4-methylpentan-2-yl)imidazol-2-ylene]}palladium(II) ethanol monosolvate Hydrogen-bond geometry (Å, º)

**Table:**

*D*—H···*A*	*D*—H	H···*A*	*D*···*A*	*D*—H···*A*
O1—H1···O3^i^	0.82	2.21	2.85 (5)	134
O1—H1···O3*A*^i^	0.82	2.13	2.80 (6)	140
O2—H2···O3	0.82	2.06	2.86 (5)	164
O2—H2···O3*A*	0.82	2.09	2.90 (7)	171
O3—H3*A*···O1^ii^	0.82	2.04	2.85 (5)	168
O3*A*—H3*AA*···O1^ii^	0.82	1.99	2.80 (6)	170

Symmetry codes: (i) *x*+1/2, −*y*+1/2, −*z*+1; (ii) *x*−1/2, −*y*+1/2, −*z*+1.
